# Injectable ion-coordinated double-network conductive hydrogel for spinal cord injury repair

**DOI:** 10.3389/fbioe.2025.1618680

**Published:** 2025-06-09

**Authors:** Huan Yu, Fan Liu, Yaorui Hu, Weikang Wan, Qing Liu, Shuai Zhou, Luping Zhang, Liming Li, Fei Huang

**Affiliations:** ^1^ School of Special Education and Rehabilitation, Binzhou Medical University, Yantai, China; ^2^ School of Sport, Exercise and Rehabilitation Sciences, University of Birmingham, Birmingham, United Kingdom; ^3^ Department of Anatomy, School of Basic Medicine, Shandong University, Jinan, Shandong, China; ^4^ School of Health and Life Sciences, University of Health and Rehabilitation Sciences, Qingdao, China; ^5^ Institute of Neurobiology, Binzhou Medical University, Yantai, China; ^6^ School of Rehabilitation Sciences and Engineering, University of Health and Rehabilitation Sciences, Qingdao, China

**Keywords:** spinal cord injury, injectable hydrogel, iron ion (Fe^3+^) coordination, double-network hydrogel, neural repair

## Abstract

The mammalian central nervous system (CNS) demonstrates a severely limited capacity for spontaneous neural regeneration after traumatic spinal cord injury (SCI). Structural repair is also highly constrained due to the inhibitory microenvironment. This inherent limitation persists throughout the recovery phase and often leads to severe motor and sensory dysfunction, profoundly impairing patients’ quality of life. Current clinical treatments, including surgical decompression, pharmacological interventions, and rehabilitation therapy, can only partially relieve symptoms. They are not enough to promote neural regeneration and functional recovery. There is an urgent need to develop novel therapeutic approaches to overcome this challenge. This study developed and created an injectable double-network conductive hydrogel, it coordinates iron ions (Fe^3+^) using dynamic Schiff base bonds and metal ion coordination. The conductive hydrogel aids in spinal cord injury repair through various mechanisms, such as reducing glial scar formation, promoting remyelination, and providing neuroprotection. This makes it an injection therapy with promising prospects for clinical translation in the field of nerve regeneration.

## 1 Introduction

Spinal cord injury (SCI) is a major cause of severe neurological dysfunction, affecting patients’ motor and sensory abilities. Although medical technology has advanced, the ability to regenerate after spinal cord injuries is still limited, this significantly lowers patients’ quality of life (Sousa et al., 2025). Current treatment methods, such as surgical and pharmacological interventions, often produce unsatisfactory results in promoting neural regeneration (Guo et al., 2023), mainly due to the lack of optimal conductive and biocompatible materials for repair (Zhu et al., 2025). Thus, it is crucial to explore new biomaterials that can enhance repair outcomes after spinal cord injury.

**SCHEME 1:**
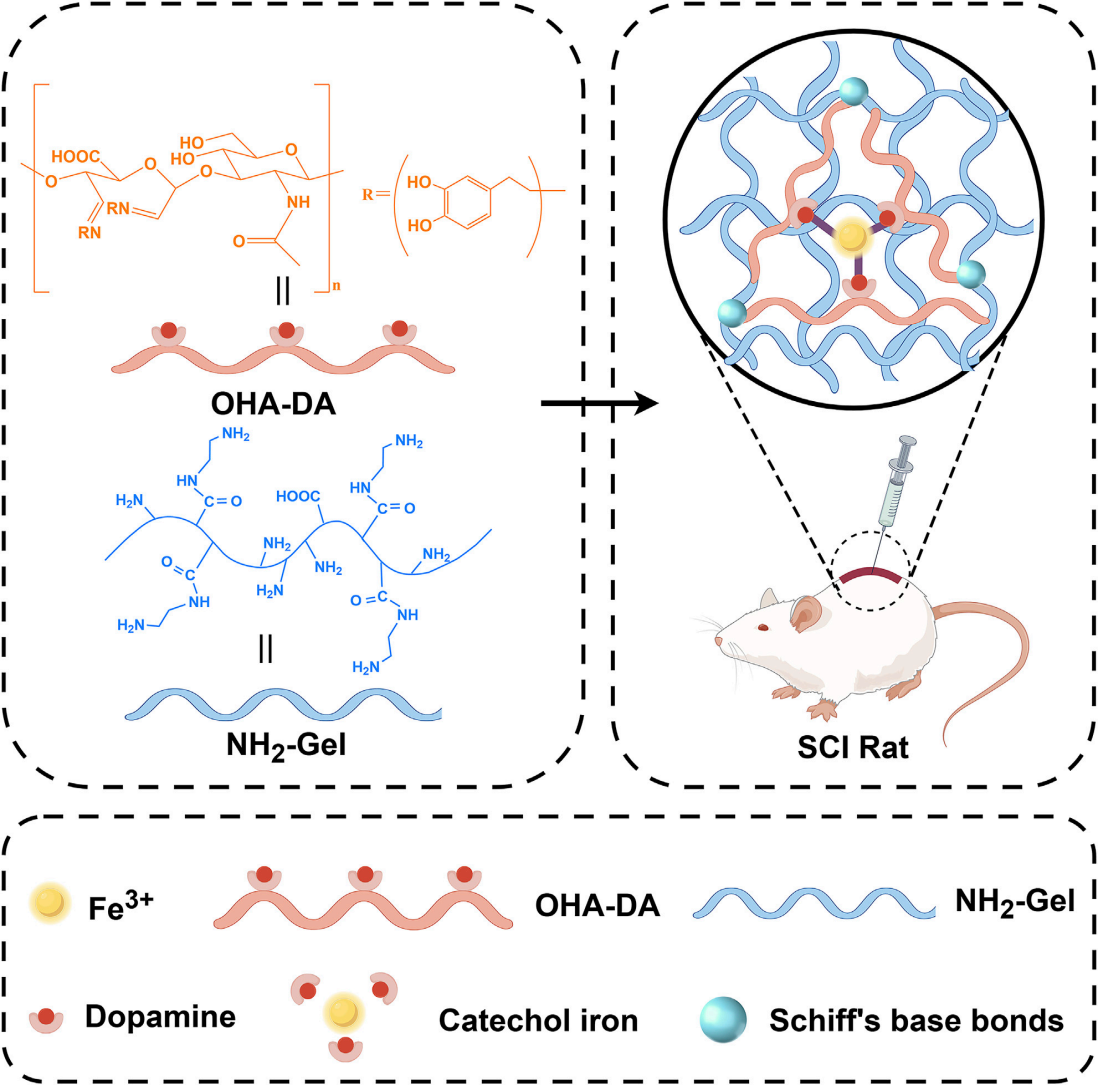
Illustration of the experimental design. An injectable double-network conductive hydrogel that coordinates iron ions (Fe^3+^) using dynamic Schiff base bonds and metal ion coordination.

Recently, researchers have recognized the importance of conductive materials in neural regeneration. Studies have shown that conductive hydrogels can provide a favorable growth environment for cells (Sahoo et al., 2025). Ferric ion-dopamine (Fe^3+^-DA) coordinated hydrogels are a type of smart hydrogel created by the dynamic coordination of Fe^3+^ with catechol groups from dopamine or its polymers. This system combines the strong adhesion and self-assembly properties of dopamine with the redox activity of ferric ions, leading to unique applications in biomedical and flexible electronics (Feng et al., 2024; Pilipenko et al., 2023; Qiao et al., 2023). The catechol groups in dopamine, or its polymer polydopamine (PDA), form dynamic coordination bonds with Fe^3+^ through chelation, creating a three-dimensional network. The catechol groups in dopamine endow the hydrogel with robust adhesion to various materials (metals, ceramics, biological tissues), mimicking the mechanism of mussel foot proteins. The Fe^3+^-catechol coordination bonds can respond to pH, redox environments (e.g., ascorbic acid, H_2_O_2_), or mechanical stress, enabling self-healing or degradation. Because of their multimodal responsiveness and biomimetic integration, Fe^3+^-dopamine coordinated hydrogels represent a leading area of research in smart materials (Huang et al., 2017). These findings provide a theoretical foundation for this study, suggesting that injectable ferric ion-coordinated double-network conductive hydrogels may play a significant role in SCI repair (Geng et al., 2023; Chi et al., 2024a). Importantly, Fe^3+^ ions themselves possess intrinsic redox activity and can participate in Fenton-like reactions, which may at first seem harmful due to reactive oxygen species (ROS) generation. However, under controlled conditions, Fe^3+^ can act as a redox modulator and even an indirect antioxidant by interacting with oxidative microenvironments (Jiang et al., 2024). In the context of SCI, where oxidative stress plays a significant role in secondary injury processes, Fe^3+^ can serve as a catalytic center for redox buffering, potentially scavenging excess ROS and mitigating oxidative damage to neural tissue. This dual functionality—structural coordination and redox modulation—makes Fe^3+^-based hydrogels particularly attractive for SCI repair, offering both mechanical support and a biochemical protective mechanism (Gao et al., 2024). However, how to effectively incorporate iron ions into hydrogels while reducing their molar concentration to minimize potential iron-related toxicity remains a challenging issue. On this basis, how to design hydrogels with injectable self-healing properties is also one of the effective means to enhance the clinical translational application of hydrogels (Li et al., 2017).

This study aims to develop a new injectable conductive hydrogel that coordinates ferric ions using Schiff base and ionic coordination bonds. The double-network structure not only ensures mechanical strength, but also enables efficient iron ion coordination, significantly reducing the required amount of iron ions. It will also assess the hydrogel’s biocompatibility and effectiveness in promoting neural regeneration through both *in vitro* and *in vivo* experiments. The research methodology involves synthesizing the hydrogel, conducting cellular experiments, and performing studies on animal models. By comprehensively considering the chemical composition, conductivity, and cellular behavioral effects of the biomaterial, this study aims to provide new insights and material support for the clinical treatment of SCI. Future research should focus on long-term outcomes and the potential for clinical translation.

## 2 Materials and methods

### 2.1 Reagents and animals

Hyaluronic acid (Jinan Huaxi Biotech); Gelatin (Shanghai Hushi); Phosphate-buffered saline (Procell); Sodium periodate (Macklin); Dopamine (Macklin); Ethylene glycol (Macklin); Ethylenediamine (Sigma); Goat anti-rabbit FITC-conjugated secondary antibody (Abbkine); Polyethylene glycol (Aladdin); N-Hydroxysuccinimide (Macklin); 1-Ethyl-3-(3-dimethylaminopropyl) carbodiimide hydrochloride (Macklin); Neutral resin (Shanghai Specimen Model Factory); Tissue embedding cassettes & paraffin wax (Shitai); CCK-8 assay kit (Biosharp); Fetal bovine serum (Procell Life Technology); Penicillin-streptomycin solution (Solarbio); Paraformaldehyde fixative (Servicebio); Trypsin (Solarbio); Modified Masson’s trichrome staining kit (Solarbio); Luxol Fast Blue myelin staining solution (Solarbio); Hematoxylin and eosin staining kit (Solarbio); Nissl staining solution (Cresyl Violet method) (Solarbio); Anti-fade mounting medium (Solarbio); Triton X-100 (Beyotime Biotechnology); 1,640 medium (Procell); Primary antibodies: Anti-GFAP (Proteintech, 60190-1-lg), Anti-NF200 (Proteintech, 60331-1-lg), Anti-MBP (Proteintech, 10458-1-AP), Anti-Tuj-1 (Proteintech, 66375-1-lg), Anti-MAP-2 (Proteintech, 17490-1-AP), Anti-ACAN (Proteintech, 13880-1-AP); Secondary antibodies: Dylight 594 Goat Anti-Rabbit IgG (Abbkine, A23420), Dylight 488 Goat Anti-Mouse IgG (Abbkine, A23210). The experimental animals were provided by Jinan Pengyue Laboratory Animal Breeding (Jinan, China), with a production license number of SCXK (Lu) 20220006. All rats were housed in a standard barrier environment at the Experimental Animal Center. The animal experimental protocol for this study received approval from the Experimental Animal Ethics Committee, referenced by the approval number Animal research (2023-401).

### 2.2 Cell culture

The PC12 cell line was provided by Shanghai Saibikang Biotechnology, and cultured according to the manufacture’s guidelines. This cell line is widely used in neurobiology research, demonstrates typical neurotrophic factor response characteristics, and serves as an *in vitro* model for studying neural cell differentiation and function.

### 2.3 Preparation of the ion-coordinated double-network injectable conductive hydrogel

The oxidized hyaluronic acid (OHA) was synthesized by dissolving 10 g hyaluronic acid in 1,000 mL deionized water, followed by reaction with 10 g sodium periodate at 25°C for 4 h in the dark. The reaction was terminated with 10 mL ethylene glycol, and the product was dialyzed (MWCO 3.5 kDa) for 3 days before lyophilization. For dopamine conjugation (OHA-DA), 8 g OHA was activated with 6.6 g EDC and 1.95 g NHS in aqueous solution (pH 5.5) for 30 min, then reacted with 4 g dopamine hydrochloride for 24 h. The resulting OHA-DA conjugate was purified via dialysis (MWCO 3.5kDa, 3 days) and lyophilized. Aminated gelatin (AG) was prepared by dissolving 5 g gelatin in 0.1 M PBS (100 mL), modifying with 10 mL ethylenediamine at pH 5 using 2.3 g EDC, and reacting at 35°C for 24 h prior to dialysis and lyophilization.

To fabricate the hydrogel, OHA-DA and AG were mixed in a 3:2 volume ratio to initiate Schiff base crosslinking. Mechanical reinforcement was achieved by adding FeCl_3_ (5 mM final concentration), which coordinated with catechol groups in OHA-DA to form secondary metal-ligand bonds. The dual-crosslinked hydrogel exhibited rapid gelation (<2 min) under physiological conditions, facilitated by the synergistic effects of covalent and coordination interactions.

### 2.4 Characterizations of the hydrogel

Observe the liquid state change in the vial. Even when tilted, the vial maintains a stable planar state for an extended period. This indicates that the system has successfully transitioned from sol to gel, resulting in a gel material with mechanical strength and structural stability. The gel samples underwent lyophilization and were subsequently examined with a scanning electron microscope (SEM). The gel was loaded into a 5 mL syringe. During the extrusion process, we observed the gel’s flow state, extrusion smoothness, and the ability to maintain a continuous flow. The hydrogel was cut to artificially create a fracture surface. The two fractured surfaces of the hydrogel were closely aligned and left standing for 5 min under natural conditions, during which we observed the extent of self-healing at the fracture surface. In addition, the healed hydrogel was gently picked up with tweezers to observe whether the two separated pieces would separate again.

### 2.5 Live/dead staining

After seeding PC12 cells (10,000 cells/dish) in confocal dishes and culturing for 24 h, the medium was removed. The hydrogel was added to the cell culture wells. After the gel solidified, complete medium was added for continued culture. At three time points of 12 h, 24 h, and 48 h, the cells were stained with a live-dead cell staining kit (White Shark). After staining, the cells were observed under a laser confocal microscope: dead cells were red, and normal cells were green.

### 2.6 CCK-8 assay

According to GB/T 16886.5 - 2017, if cell viability decreases by more than 30%, a cytotoxic reaction is indicated. In this study, the injectable conductive hydrogel was extracted at a concentration of 0.1 g/mL for 72 h. A 0.22 μm microporous membrane sterilized the extract, which was then used to prepare a complete medium containing 10% extract. PC12 cells were seeded in 96-well plates (8k-1wpl8_1 cells/well), and after 24 h of culture, the original cell culture medium in the plates was discarded. Then, the experimental samples were divided into two groups: one group continued cell culture with conventional complete medium, and the other group used the prepared complete medium containing the extract for cell culture. The survival status of PC12 cells was assessed using the CCK8 method.

### 2.7 HE staining

At postoperative week 6, comprehensive histopathological evaluation was systematically performed through hematoxylin and eosin (H&E) staining (conducted per manufacturer’s protocol) across all experimental cohorts, with particular focus on principal organ systems including the cardiac, hepatic, splenic, pulmonary, and renal tissues to characterize potential morphological alterations.

### 2.8 Animals and surgical procedures

Eighteen rats were randomly divided into three groups (n = 6): the sham operation group, the spinal cord injury group, and the spinal cord injury with hydrogel group. Both the spinal cord injury group and the spinal cord injury with hydrogel group used a complete transection model of spinal cord injury. The rats were anesthetized by intraperitoneal injection of 2% sodium pentobarbital (40 mg/kg). The hair on the back (T9-T11 region) was shaved and disinfected. Using the T10 spinous process as a reference, the skin was incised along the dorsal midline, and the muscle and fascia were dissected to expose the lamina. The T10 lamina was removed with bone rongeurs to fully expose the spinal cord. Micro-scissors were used to completely transect the spinal cord at the T9-T10 level, creating a 0.5–2.0 mm gap. After hemostasis, 50 μL of conductive hydrogel was injected into the gap. Once the hydrogel solidified, the muscle and skin were sutured in layers, followed by disinfection. Postoperatively, penicillin was administered for 7 days to prevent infection. Additionally, rats in each injury group received bladder massage three times daily to assist urination until spontaneous voiding resumed.

### 2.9 Motor function evaluation

Six weeks post-surgery, the rats were anesthetized with 2% sodium pentobarbital. Motor evoked potentials (MEPs) were then recorded. The researchers placed the rats in a prone position on the operating table and shaved their scalps. The motor area of the cerebral cortex served as the stimulation site, with an electrode connected to the trunk and a recording electrode placed on the sole of the hindlimb. Parameters were set at 500 μV, 2 m, and a stimulation intensity of 20 mA. The latency and amplitude of the potentials were recorded. The body length of the rats was measured to calculate the conduction velocity. The behavioral outcomes of the rats were assessed weekly after surgery using the Basso, Beattie, and Bresnahan (BBB) motor assessment. The rats were allowed to move freely in an open space, and their BBB motor scores were determined by observers who were blind to the experimental conditions. The motor skills of the rats were evaluated by the horizontal ladder walking test on a horizontal staircase (China Science and Technology, Beijing, China).

### 2.10 Immunofluorescence staining

The harvested spinal cord tissue was embedded and sectioned into 15 μm slices. Immunofluorescence staining was performed on the sections for neurofilament (NF; Proteintech, China), glial fibrillary acidic protein (GFAP; Proteintech, China), chondroitin sulfate proteoglycan (CSPG; Proteintech, China), neuronal Class III β-tubulin (Tuj-1; Proteintech, China), and microtubule-associated protein 2 (MAP-2; Proteintech, China). After washing, the tissue sections were incubated for 1 h with Alexa Fluor 488 and 594 conjugate secondary antibodies (Proteintech, China). The slides were then mounted with an anti-fluorescence quenching agent before imaging with a 3DHISTECH scanner (Hungary), and quantitative analysis was conducted using ImageJ software.

### 2.11 Statistical analysis

Data were analyzed using SPSS 26.0, Origin 2021, and GraphPad Prism 10, with experiments repeated ≥3 times. Normality was assessed using the Shapiro-Wilk test. Normal data are presented as mean ± SD and analyzed by one-way ANOVA and paired t-tests (significant at *P < 0.05, **P < 0.01, ***P < 0.001, ****P < 0.0001). Fiji ImageJ quantified stained sections, converting pixels to mm^2^.

## 3 Results

### 3.1 Synthesis and characterization of the injectable conductive hydrogel

**FIGURE 1:**
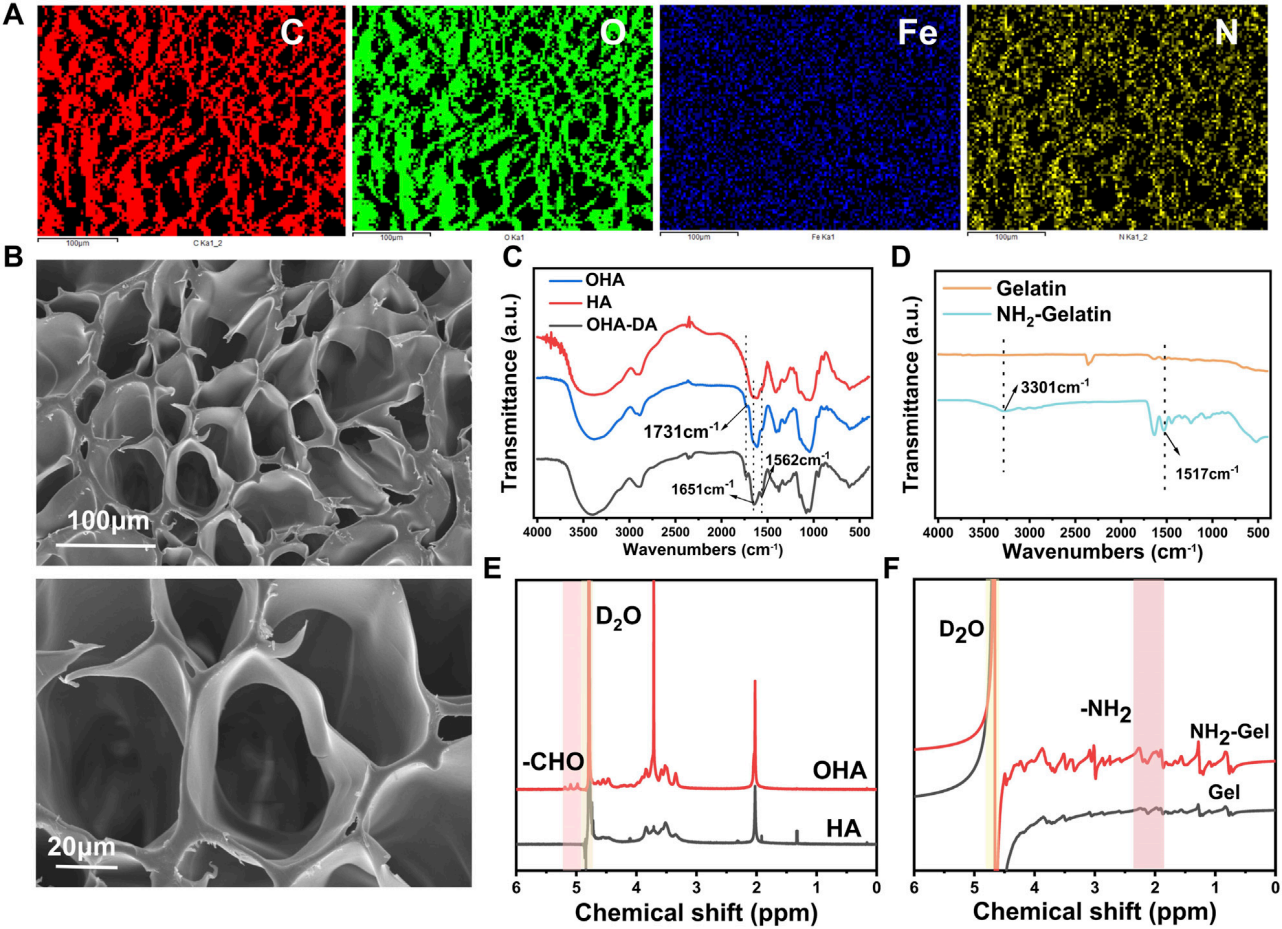
Preparation and characterization of precursor materials. **(A)** Elemental analysis of the hydrogel. **(B)** SEM image of the hydrogel. **(C)** FTIR spectra of hyaluronic acid (HA) and oxidized hyaluronic acid (OHA). **(D)** FTIR spectra of gelatin and aminated gelatin. **(E)** ^1^H NMR spectra of HA and OHA. **(F)** ^1^H NMR spectra of gelatin and aminated gelatin.

To analyze the microstructure of the hydrogel, we lyophilized the sample and examined it using a scanning electron microscope (SEM). Elemental analysis confirmed that C, O, N, and Fe were uniformly distributed throughout the gel (Figure 1A), the interior of the hydrogel had a porous structure (Figure 1B).

This study focused on oxidized hyaluronic acid (OHA), an essential part of the composite hydrogel, with sodium periodate to produce OHA (Yi et al., 2022). Fourier-transform infrared spectroscopy (FTIR) analysis showed that OHA had a distinct absorption peak at 1730–1740 cm^−1^ (Figure 1C) compared to unmodified HA. This peak is attributed to the C=O stretching vibration of aldehyde groups, confirming successful oxidation. Further characterization by proton nuclear magnetic resonance (^1^H NMR) spectroscopy showed that both OHA and HA displayed a characteristic peak at 4.79 ppm, corresponding to the proton signal of the deuterated water solvent (Figure 1E). Notably, OHA exhibited new chemical shifts in the range of 4.98–5.19 ppm, which are attributed to protons in the hemiacetal structure formed by the reaction between aldehyde groups and adjacent hydroxyl groups. These findings further validate the successful preparation of OHA.

Dopamine (DA) was grafted onto OHA chains to improve the hydrogel’s adhesive properties and conductivity. The FTIR spectrum of OHA-DA showed prominent peaks at 1,562 cm^−1^ (amide II band, N-H bending vibration) and 1,651 cm^−1^ (amide I band, C=O stretching vibration) (Figure 1C), confirming successful DA conjugation.

Ethylenediamine was used to modify gelatin, increasing its amino group density (Kushibiki et al., 2006) and enabling subsequent crosslinking reactions. FTIR analysis showed that AG, when compared to unmodified gelatin, displayed significantly stronger peaks at 1,517 cm^−1^ for N-H in-plane bending vibration and at 3,301 cm^−1^ for N-H stretching vibration (Figure 1D). Furthermore, the chemical structure of the product was further characterized by ^1^H NMR spectroscopy (Figure 1F), and the results were consistent with the FTIR analysis, confirming the successful preparation of aminated gelatin. The increased amino group content provides additional reactive sites for subsequent functionalization, facilitating further modification of the material.

These findings confirm that additional amino groups were successfully introduced into the gelatin structure.

A mixed volume ratio of OHA-DA and aminated gelatin (AG) (3:2) was used to covalently cross-link the molecules via the Schiff base reaction between amino and aldehyde groups (Wang et al., 2021), forming a preliminary three-dimensional network structure. To further improve the hydrogel’s mechanical properties, ferric trichloride was introduced as a cross-linking agent. Ferric trichloride forms stable metal-ligand bonds by coordinating iron ions with catechol groups in OHA-DA molecules. This enhances the strength of intermolecular connections and facilitates the solution-gel transition process.

**FIGURE 2:**
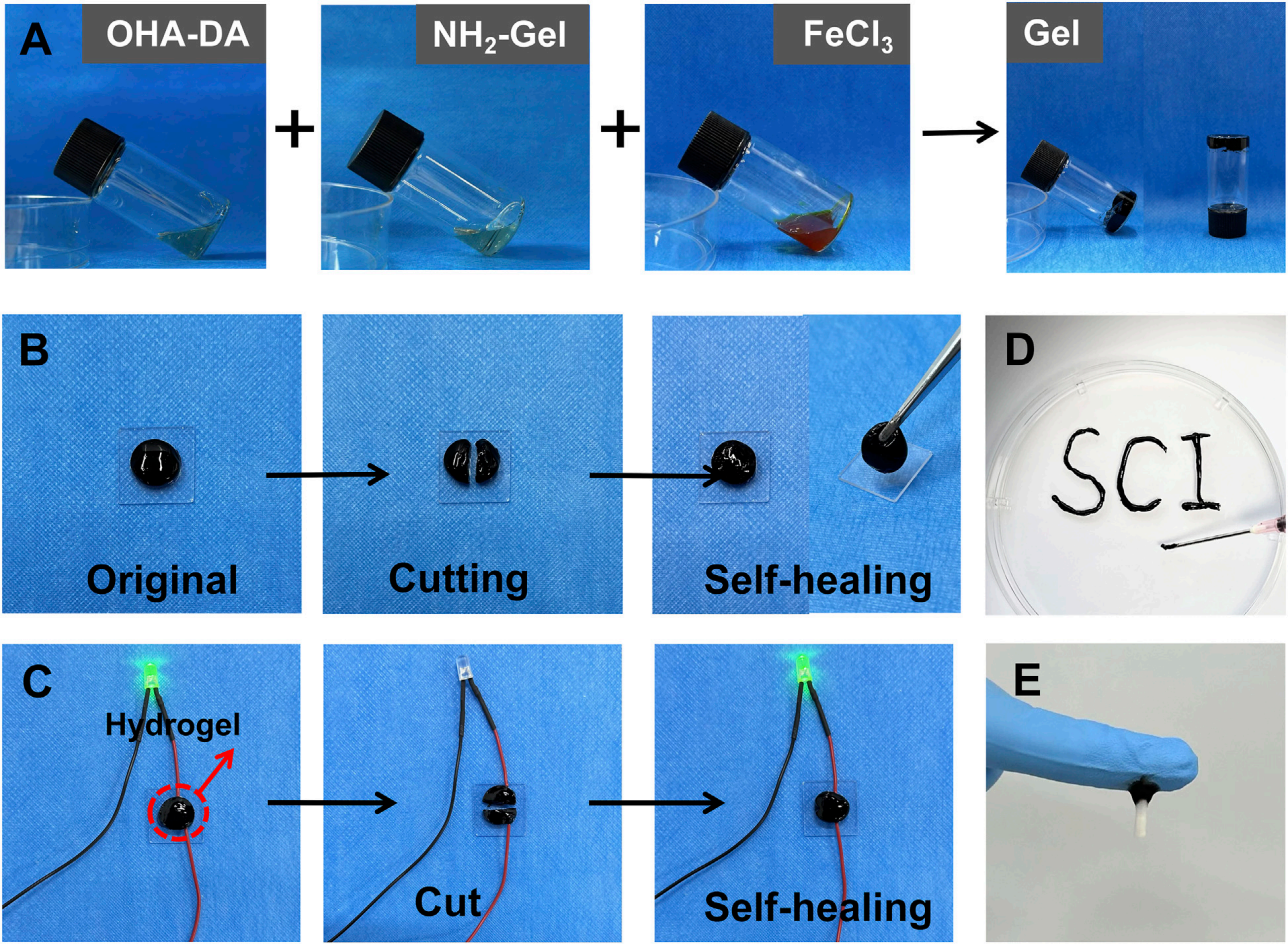
Preparation and characterization of the injectable conductive hydrogel. **(A)** Gelation process of the hydrogel. **(B)** Demonstration of the self-healing properties of the hydrogel. **(C)** Conductivity of hydrogel. **(D)** Injectability of the hydrogel. **(E)** Tissue adhesion of the hydrogel.

When the vial containing the sample was tilted slowly to observe changes in the liquid state, the system maintained a stable planar state. This stability could persist for a long time (Figure 2A). This shows that the system successfully completed the sol-gel transition, resulting in a gel material with strong mechanical properties and structural stability.

After cutting the hydrogel, the fracture surfaces were pressed together and left at room temperature for 5 min. Observations indicated that the fracture surfaces could heal spontaneously without any noticeable gaps at the interface. The mechanical properties of the healed area largely returned to their original state. Further verification through forceps clamping tests demonstrated that the healed sample did not experience additional fractures under external mechanical forces (Figure 2B), indicating that the material possesses strong self-healing properties. The self-healing ability of the hydrogel was used to demonstrate its good electrical conductivity (Figure 2C).

The hydrogel sample was placed in a 1 mL syringe, and manual pressure was used to extrude it through a 22 G needle (inner diameter 0.41 mm) (Figure 2D). The gel exhibited excellent flow characteristics during extrusion, forming a continuous and uniform stream without blockages or breakage. Notably, the extruded gel maintained its original three-dimensional network structure and morphological integrity, without significant structural damage or separation. These results indicate that the prepared hydrogel possesses excellent injectable properties, and its rheological characteristics meet the requirements for minimally invasive injection therapy. The hydrogel adhered firmly to spinal cord tissue (Figure 2E), demonstrating its excellent adhesive properties.

### 3.2 Cytotoxicity and biocompatibility of the hydrogel

**FIGURE 3:**
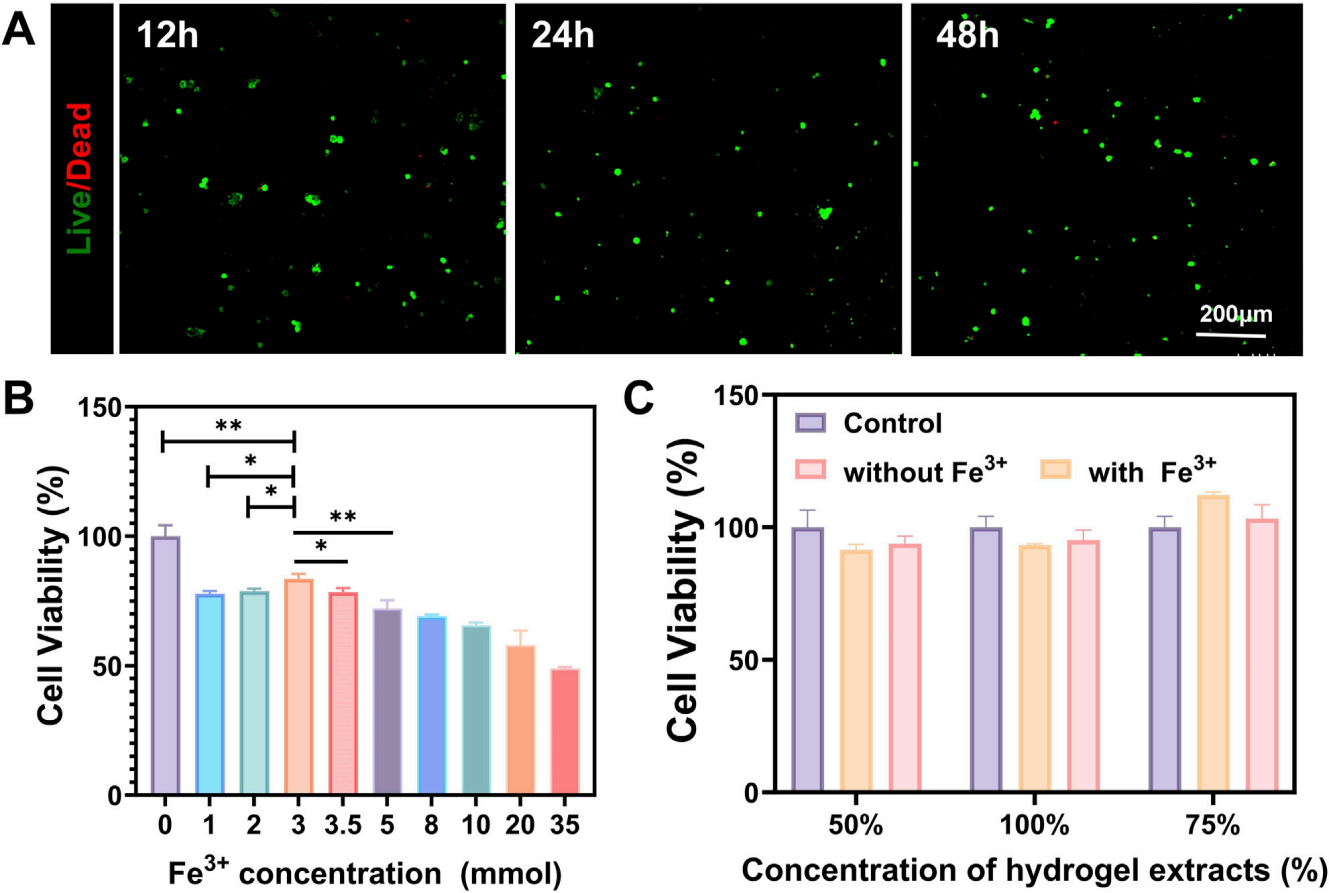
Cytotoxicity evaluation of the injectable conductive hydrogel. **(A)** Co-culture of the hydrogel with PC12 to assess cytotoxicity at 12, 24, and 48 h **(B)** CCK-8 assay to evaluate the effect of Fe^3+^ on the viability of PC12 cells **(C)** CCK-8 assay to evaluate the effect of hydrogel extracts on the viability of PC12 cells. (Data were shown as mean ± SD, *p < 0.05, **p < 0.01).

The live/dead cell staining method was used to assess cell viability, and laser confocal results showed that the number of dead cells (stained red) did not significantly increase (Figure 3A). After establishing a safe concentration of iron ions that do not harm PC12 cells (Figure 3B), we conducted a CCK-8 assay on the hydrogel extract to assess cell viability (Fan et al., 2024; Chen et al., 2022). Compared with the control group, the cell viability in each extract group did not show a significant decrease (Figure 3C). This result shows that the injectable conductive hydrogel is non-cytotoxic.

**FIGURE 4:**
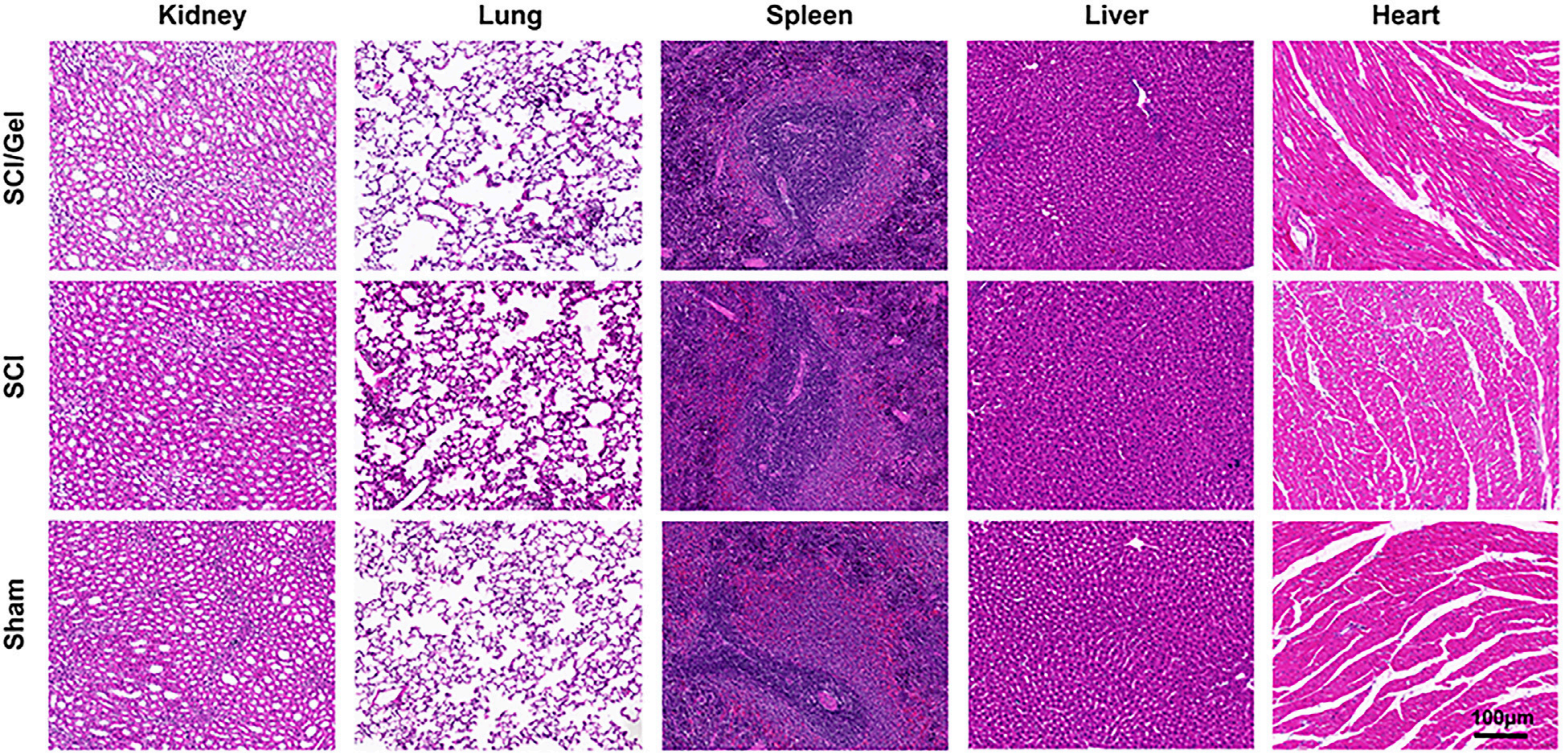
The major organs (including heart, liver, spleen, lungs and kidneys) of rats in each experimental group were analyzed by H&E staining at 8 weeks postoperatively.

When OHA is crosslinked with aminated gelatin (e.g., through the formation of Schiff bases or condensation products), the crosslinking sites can slow down the degradation rate (Mayekar and Auras, 2024). However, the material as a whole remains biodegradable by endogenous enzymes in the body. The final metabolic products are processed through normal carbohydrate and amino acid metabolic pathways and are ultimately converted into carbon dioxide, water, and urea, which are excreted from the body (Stern, 2004). Six weeks after surgery, H&E staining analysis was conducted on the main organs of rats in each experimental group to observe histological changes, including the heart, liver, spleen, lung, and kidney (Zhou et al., 2025). The results showed that, compared to the Sham group, the main organs of rats treated with conductive hydrogel were structurally intact and did not exhibit significant histological damage, with no pathological changes such as inflammatory cell infiltration, fibrosis, or necrosis observed (Figure 4). This result suggests that the conductive hydrogel is biocompatible and does not cause toxicity or damage to the main organs of the experimental animals.

### 3.3 Injectable conductive hydrogel promotes morphological and motor function recovery after spinal cord injury

**FIGURE 5:**
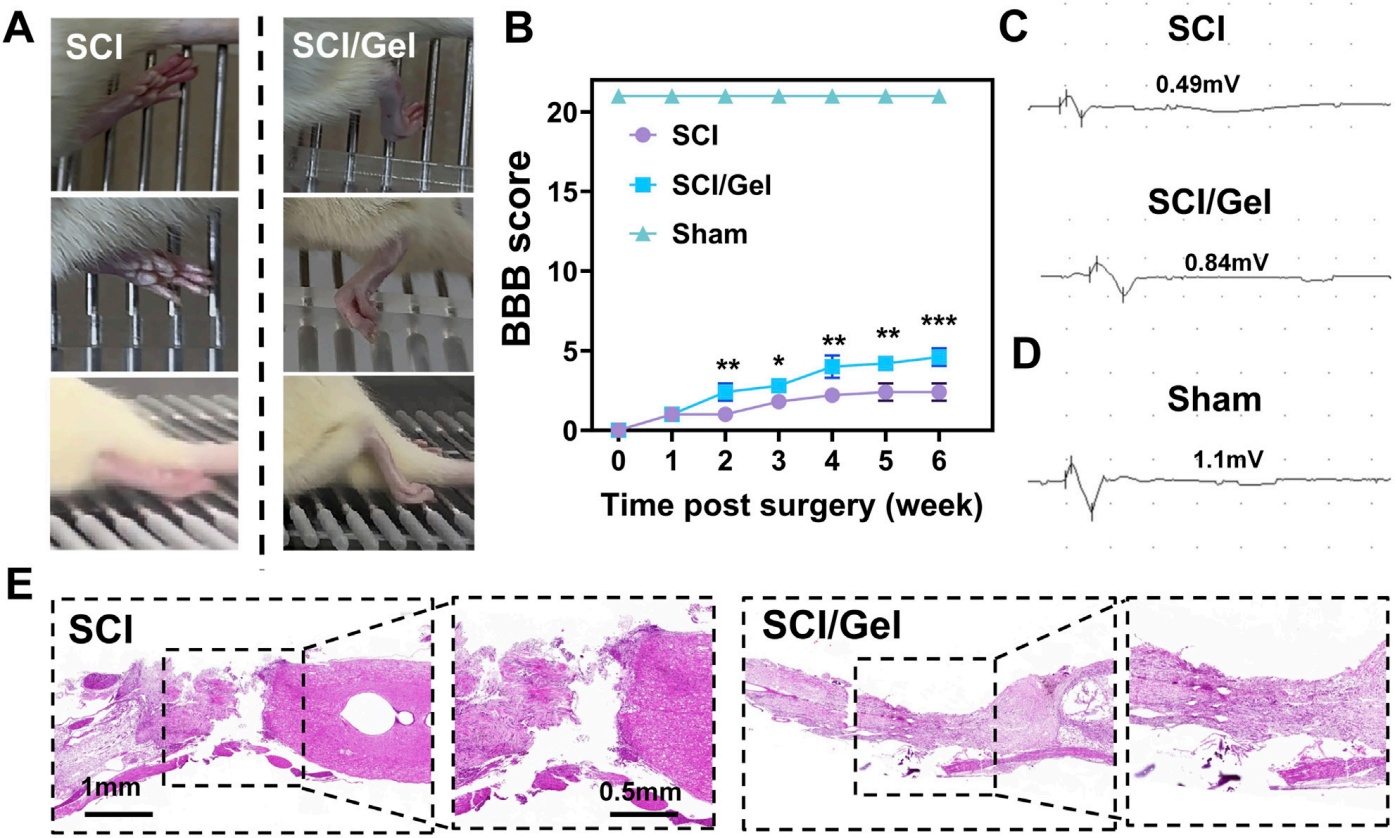
Promotion of spinal cord morphology and motor function recovery in rats after spinal cord injury by use of injectable conductive hydrogel. **(A)** Recovery of hindlimb motor function in rats. **(B)** BBB locomotor scores of rats from 1 to 6 weeks post-surgery. **(C)** Electrophysiological test results of motor evoked potentials (MEPs) in experimental groups. **(D)** Electrophysiological test results of motor evoked potentials (MEPs) in sham group. **(E)** H&E staining of spinal cord tissues from each group at 8 weeks post-surgery. (Data were shown as mean ± SD, *p < 0.05, **p < 0.01, ***p < 0.001 SCI/Gel group vs. SCI group).

Horizontal ladder climbing experiments revealed that rats in the SCI group exhibited only slight movements in their hip joints. In contrast, rats in the Gel group showed flexion movements in both their knee and ankle joints (Figure 5A).

The motor function of rats in each group was assessed using the BBB scoring method at several time points post-injury (Zhao et al., 2020). The results (Figure 5B) revealed that rats in the Gel group exhibited significantly better hindlimb motor function recovery compared to the SCI group, with the most notable improvement occurring at 4 weeks post-injury (*P* < 0.05). For motor evoked potential (MEP) detection, baseline measurements were first taken from the sham operation group, which showed an average MEP amplitude of 1.1 mV (Figure 5D). The Gel group demonstrated significant recovery of nerve conduction function, with an average MEP amplitude of 0.84 mV, while the SCI group only recorded 0.49 mV (Figure 5C).

HE staining results indicated that the SCI group had significant spinal cord cavitation and extensive abnormal cell proliferation, severely damaging the integrity of the spinal cord tissue. In contrast, the Gel group demonstrated a significantly smaller area of spinal cord cavitation and reduced abnormal cell proliferation, suggesting that this treatment effectively promotes the structural recovery of injured spinal cord tissue (Figure 5E).

### 3.4 Injectable conductive hydrogel promotes neuron survival and nerve fiber regeneration after spinal cord injury

**FIGURE 6:**
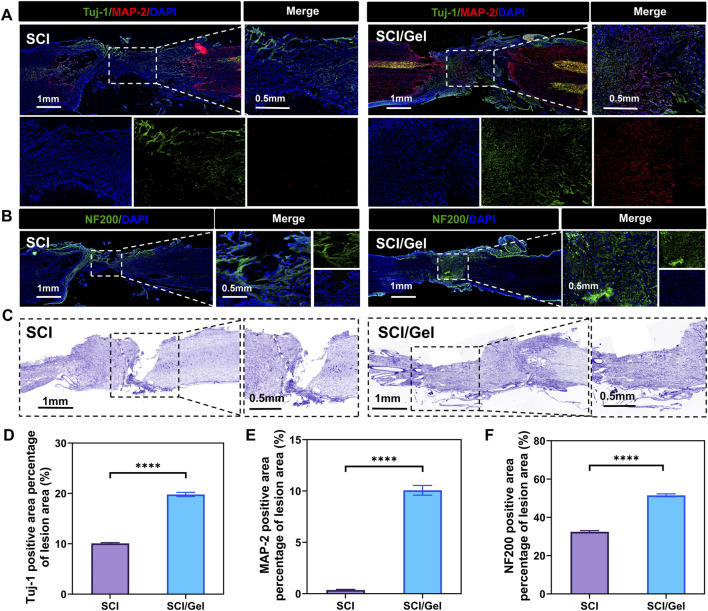
Promotion of neuronal survival after spinal cord injury by use of injectable conductive hydrogel. **(A)** Immunofluorescence staining showing the distribution of Tuj-1-positive (newborn neurons, green) and MAP-2-positive (mature neurons, red) cells. Nuclei were counterstained with DAPI (blue). **(B)** Immunofluorescence staining showing the distribution of neurofilament (NF200). Nuclei were counterstained with DAPI (blue). **(C)** Nissl staining of spinal cord tissues. **(D)** Quantitative analysis of newborn neurons (Tuj-1 positive) in the injured segments. **(E)** Quantitative analysis of mature neurons (MAP-2 positive) in the injured segments. **(F)** Quantitative analysis of neurofilament (NF200) expression. (Data were shown as mean ± SD, ****p < 0.0001 SCI/Gel group vs. SCI group).

In the conductive hydrogel treatment group, the number of newborn neurons (Tuj-1 positive) and mature neurons (MAP-2 positive) in the injury area was significantly higher than in the SCI group (Figures 6A,D,E).

Neurofilament protein staining revealed a significant reduction in neurofilament protein expression at the injury site in the SCI group. In contrast, transplantation of the conductive hydrogel markedly improved the loss of neurofilaments (Figures 6B,F).

Nissl staining results indicated that many Nissl bodies were lost in the tissues surrounding the injury area after spinal cord injury, suggesting a significant decrease in neuron survival rates. The treatment with conductive hydrogel significantly reduced the destruction of Nissl bodies, further highlighting the benefits of this strategy in neuron protection (Figure 6C).

### 3.5 Injectable conductive hydrogel alleviates glial scar hyperplasia after spinal cord injury

**FIGURE 7:**
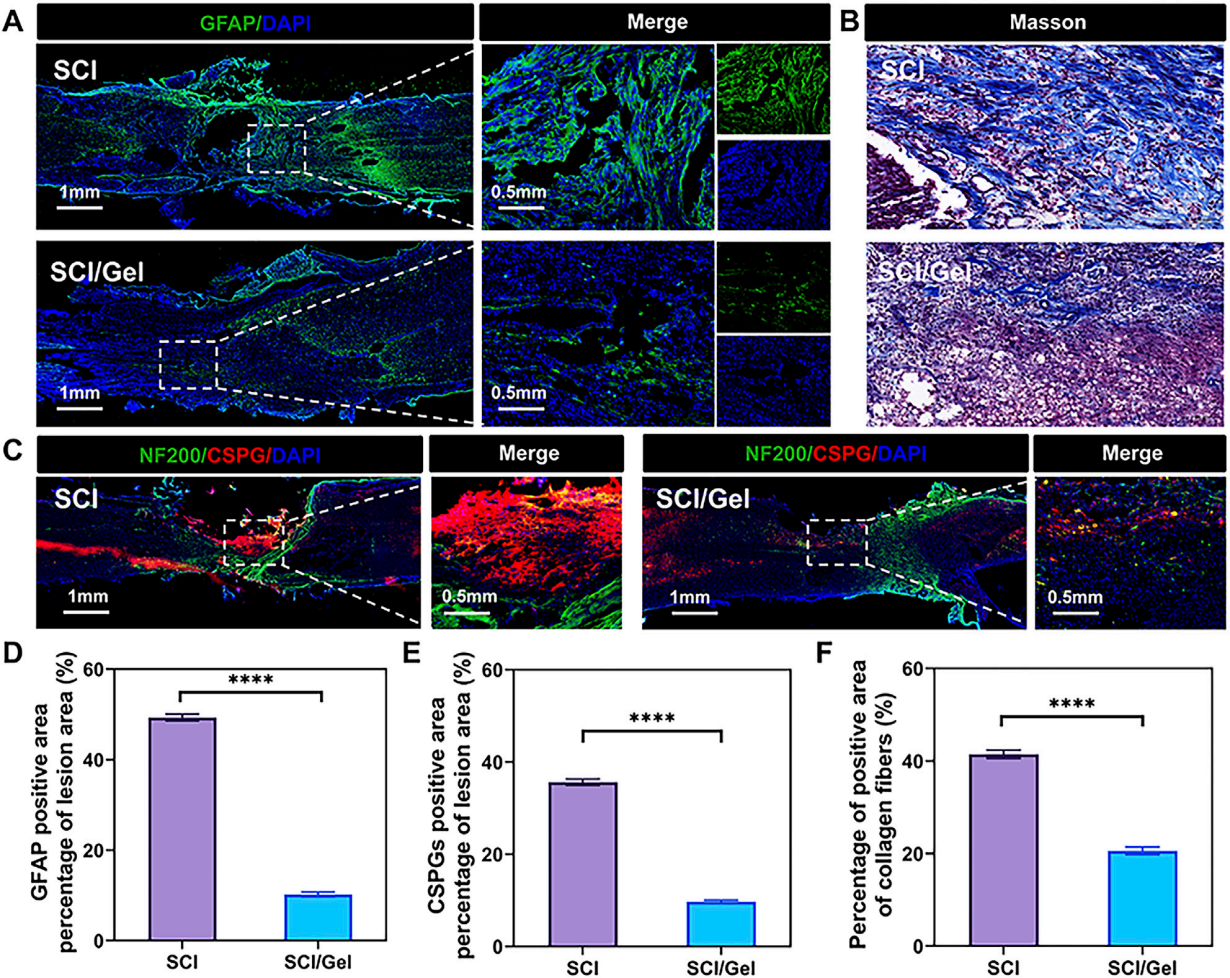
Reduction of glial scar formation after spinal cord injury by use of injectable conductive hydrogel. **(A)** Immunofluorescence staining showing the expression of the astrocyte marker glial fibrillary acidic protein (GFAP, green). **(B)** Masson staining of spinal cord sections from each group. **(C)**Immunofluorescence staining showing the co-expression of neurofilament (NF200, green) and the glial scar marker chondroitin sulfate proteoglycans (CSPGs, labeled by ACAN, red). Nuclei were counterstained with DAPI (blue). **(D)** Quantitative analysis of GFAP expression. **(E)** Quantitative analysis of CSPGs in the injured segments. **(F)** Quantitative analysis of Masson staining. (Data were shown as mean ± SD, ****p < 0.0001 SCI/Gel group vs. SCI group).

During the pathological process of spinal cord injury, the hyperplasia of glial scars is one of the key inhibitory factors hindering nerve tissue regeneration (Anjum et al., 2020). In the SCI group, many GFAP-positive astrocytes (Figures 7A,D) and ACAN-positive CSPGs (Figures 7C,E) were found around the injury area. In contrast, the transplantation of conductive hydrogel significantly reduced the formation of glial scars.

The results from Masson staining, which detects collagen, were consistent with those from immunofluorescence staining, which identifies specific proteins. In the SCI group, there was significant collagen fiber deposition along with a wide array of glial scars in the injury area. However, the group treated with conductive hydrogel exhibited reduced collagen fiber deposition (Figures 7B,F).

### 3.6 Injectable conductive hydrogel promotes remyelination after spinal cord injury

**FIGURE 8:**
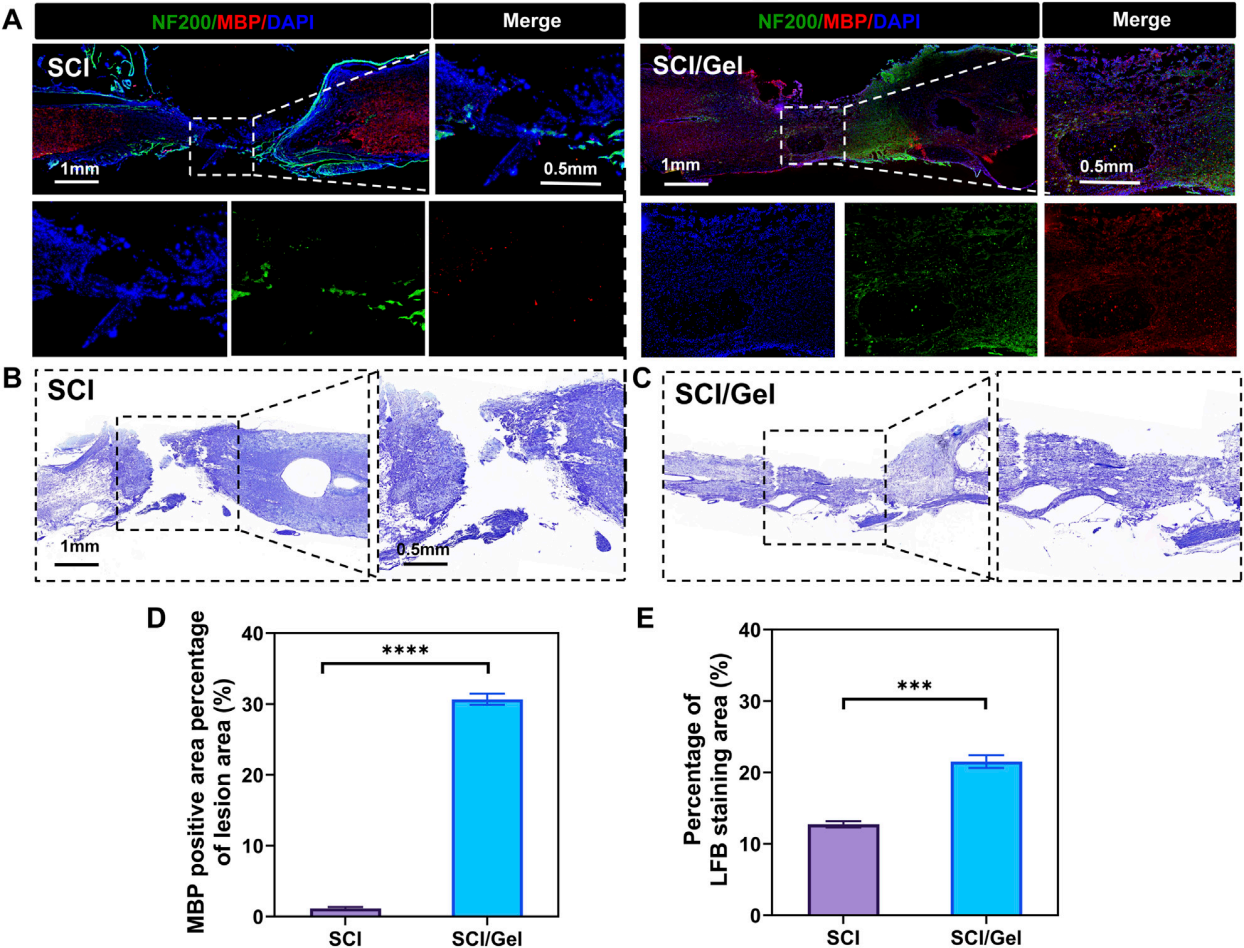
Promotion of remyelination after spinal cord injury by use of injectable conductive hydrogel. **(A)** Immunofluorescence co-staining of myelin basic protein (MBP, red) and neurofilament 200 (NF200, green) in the injury area. Nuclei were counterstained with DAPI (blue). **(B,C)** Luxol fast blue (LFB) staining of spinal cord tissues. **(D)** Quantitative analysis of MBP expression in the injured segments. **(E)** Quantitative analysis of LFB staining in spinal cord tissues. (Data were shown as mean ± SD, ***p < 0.001 ****p < 0.0001 SCI/Gel group vs. SCI group).

The multi-layered myelin sheath surrounding axons in the central nervous system enables rapid and efficient transmission of neural signals. Additionally, it provides metabolic support for neurons and helps regulate ion and water balance, adapting to the changing needs of neuronal activity (Stadelmann et al., 2019). This study observed myelin regeneration using double immunofluorescence staining for MBP and NF200, along with LFB staining. The results indicated that extensive demyelination and degeneration occurred around the injury site in the SCI group. Treatment with the conductive hydrogel significantly reduced demyelination (Figures 8A,D). The LFB staining results aligned with the immunofluorescence findings (Figures 8B,C,E).

## 4 Discussion

This study successfully developed a dual-network injectable conductive hydrogel using covalent cross-linking of ferric ion-dopamine coordination with Schiff base. Its potential application in spinal cord injury repair was confirmed through systematic *in vitro* and *in vivo* experiments. The results indicate that the hydrogel has excellent biocompatibility and injectability while significantly promoting nerve regeneration and functional recovery after spinal cord injury. The hydrogel design combines a dual-network structure with dynamic coordination bonds (Fe^3+^-catechol) and covalent cross-linking (Schiff base bonds), resulting in enhanced structural integrity and functional performance. First, the dynamic reversibility of the Fe^3+^-dopamine coordination bond provides the hydrogel with self-healing capabilities (Heidarian et al., 2022), a property that is crucial for maintaining structural integrity after long-term implantation. Second, the introduction of dopamine not only enhances the tissue adhesion of the hydrogel (Han et al., 2022; Ahmed et al., 2023; Lee et al., 2023), but also endows the material with conductivity due to the conductivity of dopamine and its combination with iron ions. This provides a bionic microenvironment for the conduction of neural electrical signals. In addition, the dual-network structure achieves a balance between mechanical properties and injectability through the synergistic effect of covalent cross-linking and ionic liganding, solving the problem of insufficient mechanical strength of the conventional hydrogels or structural instability after injection (Xu et al., 2020; Liang et al., 2024; Matyash et al., 2014). The dual-network structure also significantly reduces the amount of iron ions required for gelation compared to single iron ion coordination, thereby enhancing the biosafety of the hydrogel. Scanning electron microscopy reveals that the porous structure supports cell migration and nutrient exchange. The therapeutic effects of the hydrogel can occur through several pathways, including neuroprotection and regeneration. Hydrogel implantation significantly reduced spinal cord cavity size and promoted the survival of both newborn and mature neurons. This effect may be due to the hydrogel’s conductivity, which mimics the electrophysiological microenvironment of neural tissue and guides axon-directed growth. Additionally, the antioxidant properties of dopamine may alleviate oxidative stress from secondary injuries. Regarding glial scar inhibition, the hydrogel significantly reduced GFAP^+^ astrocyte and ACAN^+^ sulfate CSPG expression. Masson staining also confirmed reduced collagen deposition. Dynamic ligand bonding may have inhibited the release of pro-fibrotic factors by modulating the slow release of iron ions, thus breaking the vicious cycle of glial scarring; myelin regeneration was promoted: MBP and LFB staining showed a significant reduction of demyelination area in the hydrogel group, which may be related to the material’s conductivity that accelerated the migration and differentiation of chervonia cells (Wang et al., 2019; Sohn et al., 2020; Ma and Svaren, 2018), whereas the porous structure provided scaffolding support for myelin regeneration (Oh et al., 2013; You et al., 2019). Notably, the recovery of motor function (BBB score and MEP amplitude enhancement) was highly consistent with the aforementioned histological improvements, suggesting that the hydrogel achieved therapeutic efficacy through the dual action of structural repair and functional reconstruction (Cai et al., 2020; You et al., 2024; Yang et al., 2025). Although this study showed positive results, there are still unresolved issues. Specifically, the molecular mechanisms by which the hydrogel regulates neural stem cell differentiation and the combined effects of electrical stimulation and the chemical microenvironment require further investigation through transcriptomics or proteomics (Xu et al., 2021). Future research should explore minimally invasive endoscopic injection techniques to ensure the feasibility of clinical translation (Chi et al., 2024b) and consider incorporating growth factor or cells loading, such as BDNF and adipose mesenchymal stem cells, to improve therapeutic efficacy (Huang et al., 2022). For instance, in our subsequent research, we aim to incorporate piezoelectric nanoparticles into hydrogels and integrate this system with ultrasound-mediated physical rehabilitation modalities to enable wireless and site-specific electrical stimulation at the site of injury. We will further elucidate the underlying mechanisms and signaling pathways involved in the therapeutic effects of this strategy. Moreover, contingent upon feasibility, we plan to functionalize the hydrogel with stem cells or pharmacological agents, thereby fostering a multidisciplinary convergence of regenerative biology, biomaterials, and rehabilitation science to advance novel therapeutic approaches for spinal cord injury.

## 5 Conclusion

This study introduces an injectable ion-liganded dual-network conductive hydrogel that enables multi-dimensional repair after spinal cord injury, including nerve regeneration, scar inhibition, and restoration of electrophysiological function through innovative material design. Its injectability and self-repair properties enable minimally invasive treatments and inspire new clinical strategies for spinal cord injury repair. Future studies should focus on evaluating long-term safety, optimizing individualized treatment strategies, and exploring combinatorial approaches that integrate this hydrogel with stem cells, neurotrophic factors, or electrical stimulation to further enhance neural regeneration, modulate the injury microenvironment, and promote functional recovery.

## Data Availability

The raw data supporting the conclusions of this article will be made available by the authors, without undue reservation.
